# Patterned Brain Stimulation, What a Framework with Rhythmic and Noisy Components Might Tell Us about Recovery Maximization

**DOI:** 10.3389/fnhum.2013.00325

**Published:** 2013-06-28

**Authors:** Sein Schmidt, Michael Scholz, Klaus Obermayer, Stephan A. Brandt

**Affiliations:** ^1^Neurology, Vision and Motor Systems Research Group, Charité – Universitätsmedizin Berlin, Berlin, Germany; ^2^Neuronale Informationsverarbeitung, Fakultät (IV) Elektrotechnik und Informatik, Technische Universität Berlin, Berlin, Germany

**Keywords:** transcranial brain stimulation, adaptive stimulus control, synchronization, stochastic facilitation, metaplasticity, neuroplasticity, stroke rehabilitation, motor cortex

## Abstract

Brain stimulation is having remarkable impact on clinical neurology. Brain stimulation can modulate neuronal activity in functionally segregated circumscribed regions of the human brain. Polarity, frequency, and noise specific stimulation can induce specific manipulations on neural activity. In contrast to neocortical stimulation, deep-brain stimulation has become a tool that can dramatically improve the impact clinicians can possibly have on movement disorders. In contrast, neocortical brain stimulation is proving to be remarkably susceptible to intrinsic brain-states. Although evidence is accumulating that brain stimulation can facilitate recovery processes in patients with cerebral stroke, the high variability of results impedes successful clinical implementation. Interestingly, recent data in healthy subjects suggests that brain-state dependent patterned stimulation might help resolve some of the intrinsic variability found in previous studies. In parallel, other studies suggest that noisy “stochastic resonance” (SR)-like processes are a non-negligible component in non-invasive brain stimulation studies. The hypothesis developed in this manuscript is that stimulation patterning with noisy and oscillatory components will help patients recover from stroke related deficits more reliably. To address this hypothesis we focus on two factors common to both neural computation (intrinsic variables) as well as brain stimulation (extrinsic variables): noise and oscillation. We review diverse theoretical and experimental evidence that demonstrates that subject-function specific brain-states are associated with specific oscillatory activity patterns. These states are transient and can be maintained by noisy processes. The resulting control procedures can resemble homeostatic or SR processes. In this context we try to extend awareness for inter-individual differences and the use of individualized stimulation in the recovery maximization of stroke patients.

## Introduction

With 65,133 deaths in 2006, stroke ranked third place among all causes of death (7.9%) in Germany. The annual stroke incidence rate is approximately 120 per 100,000 adjusted to the European population (European Registers of Stroke et al., 2009), leading to about 100,000 new strokes in Germany per year and leaving about one million people with residual impairments. Globally an estimated 30.7 million people have survived stroke and stroke is considered to be the primary cause of disability (Norrving and Kissela, 2013). Spontaneous recovery after a cerebral insult is generally insufficient, the success of post-stroke rehabilitation is highly limited and novel therapeutic options are lacking (Wolfe et al., 2011). About 42% of stroke patients require rehabilitative and 25% inpatient care (Toschke et al., 2010) with paresis being one of the most disabling and important factors in stroke outcome (Toschke et al., 2010).

Increasing evidence summarized in a number of independent editorials and reviews supports the use of non-invasive brain stimulation (NBS) to maximize the speed and success of spontaneous recovery processes after stroke (Hallett, 2005; Talelli and Rothwell, 2006; Hummel et al., 2008; Nitsche et al., 2008; Nowak et al., 2010; Bastani and Jaberzadeh, 2012). Yet, in contrast to the successful clinical implementation of deep-brain stimulation in the therapy of movement disorders, it seems evident that NBS therapy regimes are still experimental and will require further robust refinement before entering standard clinical care (Hallett, 2005; Hummel et al., 2008; Nitsche et al., 2008; Plow et al., 2009; Grefkes and Fink, 2012). The lack of robust results and necessity of further refinement could be due to various factors. For example, the stimulus paradigm, the optimal time point, the duration, the hemisphere, and region or regions of stimulation as well as how these factors might interact with the level of impairment, type of impairment, patient age and the dynamics of inhibition, and excitation reflecting different stages of recovery processes (Dimyan and Cohen, 2011; Schulz et al., 2013). Conversely, there is a wide consensus that promising developments are the combination of NBS with subject or recovery specific factors such as physiotherapy and sensory input (Dimyan and Cohen, 2011; Schulz et al., 2013) or brain-computer interface decoding of sensorimotor brain-states (Jackson and Zimmermann, 2012). This demonstrates that the understanding of the underlying mechanisms of NBS is still incomplete and that the manipulation of subject and function specific factors will gain on importance for recovery maximization in neurobiological diseases (see also Plow et al., 2009). Interestingly, recent findings suggest that a reappraisal of the neurophysiological mechanisms of NBS with regards to their frequency components might help resolve the challenge of subject-function specific stimulation. Here, patterned stimulation is providing promising results in brain-state^1^ specific modulations in cognitive neuroscience (Thut et al., 2012). In parallel, noisy or “stochastic resonance” (SR)-like processes have been suggested to provide a common framework component that can reconcile previous contradictory findings in NBS studies (Harris et al., 2008; Schwarzkopf et al., 2011). Together, these findings suggest that oscillation and noise are both components in a common framework that require further investigation. Thus we review theoretical and experimental evidence to address the following questions. Which patterns of stimulation might optimally modify brain-states associated with motor recovery in stroke patients? What evidence is there that noise induced resonance effects are a component? This being established, we will argue that a closed-loop optimization of a state-variable (similar to brain-computer interface decoding) is a promising approach to optimally configure patterned NBS paradigms.

## Neural Oscillations and Noise

### Intrinsic sources of oscillations

The first of many band-width confined EEG pattern’s reflecting brain rhythms described were α (∼8–12 Hz) waves (Berger, 1929). Subsequent research found that these EEG pattern’s reflect neural oscillations in a system of dynamically coupled brain oscillators, each defined by the intrinsic ability of single neurons to resonate at specific frequencies, the physical architecture of a given neuronal network, and its computational restraints due to axon conductance and synaptic delay (Buzsaki and Draguhn, 2004). An oscillator (single neurons and networks) is predominantly characterized by its eigenfrequency, showing its ability to resonate in a sharply tuned frequency band. In neurons, the required band-pass characteristic is delineated by neuronal membrane capacitance and leakage currents (forming a low-pass filter) and neuron specific voltage-gated currents (acting as a high-pass filter), with which it responds to a significant input (Llinas, 1988; Hodgkin and Huxley, 1990; Hutcheon and Yarom, 2000; Augustin et al., 2013).

### Oscillations are associated with specific information transmission

**Figure 1:**
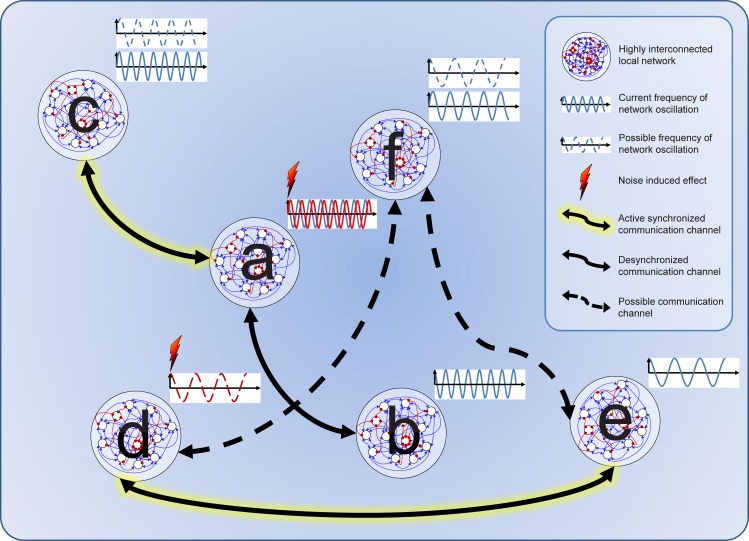
**Cartoon of network dynamics showing noise induced switching of transient communication channels**. At start, a network is synchronized with network b. Noise than induces a phase shift in b, thus desynchronizing this channel and synchronizing a with c. Noise can also induce oscillation in d synchronizing it with e. F Could principally also synchronize with d and e, but is currently in its different oscillation mode.

The adaptive tuning of high- and low-pass filtering enables the brain to subsequently construct and maintain a multitude of different band-width-confined communication channels associated with specific functions spanning from the amplification of weak signals over the reduction of environmental input to the focusing on a specific input (Buzsaki and Draguhn, 2004). The brain architecture encoding this communication is understood to utilize highly interconnected local neuron arrays (hypercolumns) sparsely interconnected by long-range connections. Within this “small-world” architecture (Watts and Strogatz, 1998; Sporns et al., 2004; Yu et al., 2008), the most energy efficient mechanism to flexibly integrate multiple segregated neuron assemblies is transient synchronization by oscillation (i.e., phase-locking Engel et al., 2001; see Figure 1). This highly dynamic integrative process is viewed as the mechanism linking single neuron activity to motor output and behaviors (see in review Buzsaki and Draguhn, 2004).

### Different bandwidths are associated with different aspects of motor function

Neuronal oscillators resonate between 0.05 and 500 Hz (Buzsaki and Draguhn, 2004), macroscopic brain oscillations synchronize between up to at least 200 Hz (Grenier et al., 2001). In the motor system the most evident bandwidth is the β (or high rolandic μ) rhythm. 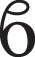-oscillations are understood to facilitate long-range interactions between distant network nodes (Donner and Siegel, 2011) signaling predominantly the maintenance of *status quo* or steady-motor output in a sensorimotor behavioral loop^2^(Farmer et al., 1993; Salenius and Hari, 2003; Baker, 2007; Faisal et al., 2008; Engel and Fries, 2010). In contrast, γ-oscillations are understood to reflect more focal (Donner and Siegel, 2011) neural activity related to contraction strength (Salenius and Hari, 2003), attention processes, movement preparation (Pfurtscheller et al., 2003) and execution (Baker, 2007; Engel and Fries, 2010; van Wijk et al., 2012), faster reaction times (Joundi et al., 2012), and heightened cortico-fugal output efficiency (Schoffelen et al., 2005) as well as motor learning (Brown, 2000; van Wijk et al., 2012). Recent evidence shows that non-invasive stimulatory entrainment of β-oscillations slows whereas entrainment of γ-oscillations expedites motor performance in simple tasks (Joundi et al., 2012). Motor performance in more complex tasks, requiring sensorimotor short-term memory integration, was susceptible to phase-dependent entrainment of θ- but not β-oscillations in a frontoparietal network (Polania et al., 2012). Thus a common small-world like wired framework (Watts and Strogatz, 1998) can be understood to confer frequency-division multiplex^3^ coherent (Engel et al., 2001) information from neural oscillator assemblies between distant motor nodes to form brain-state’s translating neural activity into simple or complex motor tasks and behavior (Brown and Marsden, 2001; Varela et al., 2001; Brown, 2003; Schoffelen et al., 2005; Engel and Fries, 2010; Donner and Siegel, 2011).

### The role of noise

Why should one include noise when utilizing NBS to improve restitution? The better question might be: “could we afford to exclude it”? To address these questions we have to define what exactly is meant by the term “neural noise,” which: (i) sums up all neural activity which could not directly be associated with a specific function but may be part of the signal instead of random noise (Stein et al., 2005), (ii) is not constant, i.e., the level of noise differs with respect to brain-states (Misic et al., 2010) and extrinsic input (Harris and Wolpert, 1998), and (iii) is always present, throughout all systems in the whole brain (Shadlen and Newsome, 1994; Ermentrout et al., 2008; Clarke, 2012). It is therefore important to understand if the brain is just robust against noise – which is undisputed – or if it utilizes noise, meaning that neural noise is an important and necessary system ingredient (Shadlen and Newsome, 1994; Friston, 1997; Lindner, 2004; Stein et al., 2005; Sejnowski and Paulsen, 2006; Ermentrout et al., 2008; McDonnell and Ward, 2011). Conjecturing that the brain is a system utilizing distinct frequencies from null to above 200 Hz as possible communication channels and that the selection of “active” channels is done via phase-locking, the question is which mechanism putatively acts as the “switch”? To put it more blatantly: could neural noise (in the notion of a complex, not understood, and non-random mechanism) act as the controller of communications in the brain (Horsthemke and Lefever, 1984; Neiman et al., 1999; Sosnovtseva et al., 2001; Misic et al., 2010)?

### Intrinsic sources of noise

In the behavioral loop noise is generated at multiple stages: i.e., sensory, cellular, and motor noise. The central cellular noise can be decomposed into electrical and synaptic noise (Calvin and Stevens, 1968). In the absence of input, electrical noise is produced by random opening and closing of voltage- or ligand-gated ion channels (White et al., 2000) or changes in membrane resistance (Yarom and Hounsgaard, 2011) as well as cross-over talk due to the activity of nearby neurons (ephaptic couplings Debanne, 2004), after extensive electrical signaling or spillover of neurotransmitters). This noise can affect the initiation (Bryant and Segundo, 1976), timing (Mainen and Sejnowski, 1995; Schneidman et al., 1998), and propagation (Diba et al., 2006) of action potentials in the neural network. In contrast, synaptic noise is associated with an intense bombardment from thousands of synapses and a large number of unresolved mechanisms (Moss et al., 2004) that provide a sigmoidal transition from attenuating and facilitating signal transmission in a neural network (Destexhe and Contreras, 2006). Networks of neurons dynamically control and utilize noise effects by homeostatic adjusting of local synaptic strengths and ion-channel expression or neuromodulator release as well as global wiring strategies (Faisal et al., 2008).

### Computational modeling exploits putative functions of noise

Stochastic resonance (Benzi et al., 1981) is one of the most widely investigated phenomena associated with attenuation and amplification by neural noise (Hanggi, 2002; Moss et al., 2004; Ward et al., 2010; Durrant et al., 2011; Mejias and Torres, 2011; Schwarzkopf et al., 2011; Torres et al., 2011). Traditionally defined SR describes sub-threshold signal transmission in the presence of an optimal noise level in dynamic non-linear excitable systems but has been recently extended to “stochastic facilitation” (McDonnell and Abbott, 2009) relaxing the strict sub-threshold signal constrictions in SR to be of (even) more practical use. Although such reliable signal transmission is an important factor, it becomes even more interesting when observing the complex dynamics of excitable systems. First of all, it’s important to note, that noise-free systems of coupled oscillators are generally unstable (Strogatz and Mirollo, 1991). In the presence of noise, the state space of these systems becomes richer, introducing new oscillatory states (Horsthemke and Lefever, 1984; Timme et al., 2002; Ostojic et al., 2009). It has been shown that noise: (i) can “switch” between these states (Neiman et al., 1999; Bascones et al., 2002; Misic et al., 2010), (ii) induces oscillations itself (Zhou et al., 2003; Ermentrout et al., 2008; Ghosh et al., 2008), and (iii) enhances phase synchronization (Neiman et al., 1999) as well as de-synchronization (Kurrer and Schulten, 1995). When considering realistically large coupled excitable systems of model neurons, the presence of noise leads to a clustering of frequencies (Postnov et al., 2001; Sosnovtseva et al., 2001; Brunel and Hansel, 2006; Deco et al., 2009), i.e., neurons form groups characterized by (almost) the same “stochastic eigenfrequency.” The number of such clusters strongly depends on the distribution interval of coupling, the larger the coupling the less clusters form. Interestingly a relaxed notion of phase-locking is sufficient for this phenomenon to occur (Sosnovtseva et al., 2001). Kurrer and Schulten (1995) have shown, that the role of noise in a system of coupled excitable systems is twofold: in the low-noise transition, noise induces synchronicity, while in the high-noise transition it leads to a loss of coherency (de-synchronization), following a sigmoid transition function between synchronization and de-synchronization. There is a large number of experimental data supporting this model in different systems throughout the whole brain and various species (Bezrukov and Vodyanoy, 1995; Gutkin et al., 2009). Considering these results, it seems not too farfetched to look upon noise as a powerful control mechanism over neural computation (see also McDonnell and Ward, 2011) for an elaboration on “stochastic facilitation.”

Thus, under the assumption that a general framework exists that sufficiently explains NBS related variability, these modeling results and experimental findings show that neural noise is not just there but in contrast has a very prominent role in controlling neural communication. This control manifests through the induction of oscillations and phase synchronization and de-synchronization, thus providing a switching agent between transient oscillatory brain-states (Destexhe and Contreras, 2006) known to require activity dependent control mechanisms (Bienenstock et al., 1982; Abraham, 2008).

## Non-Invasive Brain Stimulation

### Non-invasive brain stimulation, mode-of-action

Transcranial brain stimulation encompasses various tools that can induce long-term neural plastic changes (Ahissar et al., 1992; Huang et al., 2005; Paulus, 2011), modulate neural noise (Harris et al., 2008; Terney et al., 2008), and entrain cortical neuronal assemblies to frequency-specific oscillatory input (Zaehle et al., 2010; Thut et al., 2012). For example, transcranial direct current stimulation (tDCS) effects are typically related to elevated/decreased firing rates in neuronal structures (Creuzfeldt and Struck, 1962; Bindman et al., 1964) with after-effects analogous to long-term potentiation (LTP) and depression (LTD) (Nitsche and Paulus, 2000). Transcranial alternating current stimulation (tACS) is understood to be associated with the modulation of neural oscillations associated with powerful and coherent synchronization in cortico-thalamo-cortical loops (Terney et al., 2008), conducive of information processing and storage (Sejnowski and Paulsen, 2006). Transcranial random noise stimulation (tRNS) effects could possibly be associated with various mechanisms, e.g., high frequency oscillations (80–200 Hz ripples) related to plasticity processes (Grenier et al., 2001; Ponomarenko et al., 2008), repetitive opening of Na^+^ channels (Schoen and Fromherz, 2008) or elevated firing rates due to noisy inputs related to SR (Moss et al., 2004; Antal et al., 2008). Repetitive transcranial magnetic stimulation (rTMS) with longer trains of low (0.2–1 Hz) or high (>5 Hz) frequency stimulation have been shown to cause longer-lasting decrease or increase in brain excitability, respectively (Pascual-Leone et al., 1994; Khedr et al., 2007). These effects can be expedited with repetitive (5 Hz “theta”) trains of short high frequency (∼50 Hz) bursts delivered either intermittently or continuously (Theta-burst stimulation, Huang et al., 2005). The carry-over effects induced by rTMS are typically also understood to reflect LTP- and LTD-like effects, although the variability of findings provide some uncertainty about the underlying mechanisms (Huang et al., 2005; Paulus, 2005; Pell et al., 2011). Interestingly, recent studies suggest noise as a missing component in a common framework of TMS that can successfully reconcile previous seemingly contradictory findings of both impairment and facilitation (Schwarzkopf et al., 2011). Similarly, recent stimulation studies provide evidence that stimulation-frequency and -state dependencies can help reconcile inhibitory and facilitatory (Huang et al., 2005; Antal et al., 2007; Rothkegel et al., 2010) as well as subject-function specific variability (Schmidt and Lee, 2005; Plow et al., 2009; Thut and Pascual-Leone, 2010). In summary, both weak- and high-voltage transcranial stimulation can in some cases induce LTD- and LTP-like effects (Huang et al., 2005; Paulus, 2011), yet the underlying mechanisms are unclear and a common framework unifying oscillatory and noisy action-modes has not been studied.

### Oscillatory multiplex stimulation opens “communication channels”

It is well established that NBS is related to brain-states associated with brain functions or dysfunctions amenable to specific modulation by NBS (Siebner et al., 2010; Thut et al., 2012). Rhythmic NBS can assert an instantaneous and transient modulation (synchronize or desynchronize) of ongoing brain oscillations. Longer application will assert longer modulation, entrainment of specific frequencies and after-effects (Moliadze et al., 2010a; Zaehle et al., 2010; Joundi et al., 2012; Thut et al., 2012; Schmidt et al., 2013). Yet the situation seems more complex, as brain-states are characterized by a weighted-mixture of multiple coherent oscillatory processes (Engel et al., 2001; Varela et al., 2001) and modification might imply stimulation in multiple bandwidths. In line with this notion, recent data could demonstrate that multiplex patterned stimulation modulates specific brain-states resulting in specific behavior modifications (Thut et al., 2012). These results have led to the notion that the patterning of NBS can open communication channels defining (electrophysiological) specific finger-prints^4^ of brain function and dysfunction (Thut et al., 2012).

### Noisy stimulation, a “control mechanism” in a common framework

In contrast to oscillatory stimulation relatively few studies have addressed noisy or stochastic stimulation. In support of the notion that noise plays a specific role in brain computations and stimulation, tRNS has been shown to induce carry-over effects suggestive of neural adaptation (Moliadze et al., 2010b, 2012; Schwarzkopf et al., 2011). Yet, bi-directional manipulations well established for tDCS were lacking for tRNS. Initial, possibly misleadingly, tRNS results found only positive carry-over effects (Antal et al., 2008; Terney et al., 2008). Otherwise, both tDCS and tRNS were understood to modulate the firing rate probability possibly by membrane de-polarization (Paulus, 2011). These findings were possibly rectified by recent comparison of tDCS and tRNS, which show an “unexpected” similarity between tDCS and tRNS (Moliadze et al., 2010b) and stimulus strength dependent bi-directional effects also for tRNS (Moliadze et al., 2012). Similar to low-voltage tRNS, high-voltage TMS results might be related to the induction of noisy processes in the brain (Harris et al., 2008; Schwarzkopf et al., 2011). For example, the finding of stimulus (noise) strength dependent bi-directional results were suggested not only to reflect SR processes but also to provide a missing component in a framework that can reconcile previous apparently contradictory findings (Schwarzkopf et al., 2011). Despite emerging evidence for possible similarities between different forms of NBS (strength dependent bi-directional carry-over effects) it is still unresolved how exactly noisy stimulation might affect brain computations.

Given computational evidence that neural noise is related to sigmoidal bi-directional control mechanisms (Destexhe and Contreras, 2006), mode-of-action evidence that both tDCS and tRNS modify firing rates and recent evidence that both TMS and tRNS induce noise strength dependent bi-directional effects, we argue that noisy processes are indicative of metaplastic homeostatic control mechanisms (see also Hamada et al., 2008). This being said, the Bienenstock, Cooper, and Munro model (BCM) is currently considered to be the most influential theory of synaptic plasticity (Bienenstock et al., 1982). The vital extension is a sigmoidal metaplastic control component that can potentiate and depress synaptic plasticity and contain runaway potentiation or depression (Abraham, 2008). An association between homeostatic metaplasticity, the BCM model and NBS driven manipulations has been established in various studies with various forms of high- and low-voltage stimulation (Gentner et al., 2008; Hamada et al., 2008; Ziemann and Siebner, 2008; Jung and Ziemann, 2009; Fricke et al., 2011). Similarly well established is the association of noisy processes with a sigmoidal transformation of all-or-none spike-probability response curves and control mechanisms that are either highly beneficial or detrimental for network computations (Destexhe and Contreras, 2006). A noise dependent homeostatic component could reconcile previous contradictory NBS findings from various NBS methods. For example, it would explain the finding of only positive effects after weak (<1 mA) tRNS (Terney et al., 2008; Chaieb et al., 2011), bi-directional results for weaker and stronger TMS (Schwarzkopf et al., 2011) or tRNS (Moliadze et al., 2012), and the unexpected finding of similar effects for tDCS and tRNS in direct comparison (Moliadze et al., 2010b).

### Patterned brain stimulation studies, what might rhythmic and noisy stimulation tell us about restitution?

In stroke patients, clinical scores are the gold-standard to monitor the success of recovery (Cramer et al., 2011; Schulz et al., 2013). These scores are reliable, modality-specific, and time demanding. In NBS studies, changes in motor performance are most often quantified with behavioral measures, which typically capture fine motor skills that use smaller muscles to perform discrete precise tasks (Schmidt and Lee, 2005). These measures result in quantifiable results related to finger-tapping speed, muscle fatigue, pinch-strength or simple-, choice-, and serial-reaction times (Leocani et al., 1997, 2000; Manganotti et al., 1998). In comparison neurophysiologic measures (e.g., neural oscillations or cortico-spinal coupling) are fast, continuous, modality-specific (Brown and Marsden, 2001) and reflect fine motor skills (Halliday et al., 1998; Engel and Fries, 2010), motor learning processes (van Wijk et al., 2012), and can found in the absence or independent of behavioral measures (Laureys et al., 2001; Brown, 2003; Schoffelen et al., 2005; Fang et al., 2009; Donner and Siegel, 2011). They can also capture the dynamics of recovery processes (Brown, 2003; Strens et al., 2004; Swayne et al., 2008; Fang et al., 2009; Engel and Fries, 2010; Thut et al., 2012) and have successfully controlled brain-computer interfaces supporting patients-driven rehabilitation in neurological disease (Jackson and Zimmermann, 2012). Within this framework recovery can be conceptualized as the reestablishment of functional from dysfunctional brain-states (Hummel et al., 2008; Jackson and Zimmermann, 2012; Thut et al., 2012) and recovery maximization as the facilitation of spontaneous (coherent) neural processes driving the transition between these two states (Hummel et al., 2008; Jackson and Zimmermann, 2012; Thut et al., 2012). Thus, although data is missing to detail the characteristics of transitional states, there is ample evidence that post-stroke recovery processes should be amenable to patterned stimulation and that patterned stimulation should provide more specific stimulation effects.

It is well established that NBS can modify cortical excitability and that the manipulation of cortical excitability can maximize recovery processes (Hallett, 2005; Talelli and Rothwell, 2006; Nitsche et al., 2008) with a reported 8–30% range in functional improvement in patients recovering from stroke (Hummel et al., 2008). A classic example for NBS induced recovery maximization, is the therapeutic reduction of pathological contralesional hyperexcitability associated (transcallosal) with stroke induced ipsilesional hypoexcitability. This interhemispheric “disinhibition” phenomenon has been shown to be negatively and its therapeutic reduction with NBS positively associated with a patient’s outcome (Murase et al., 2004; Hummel et al., 2008). Similarly, the facilitation of ipsilesional excitability (Kim et al., 2006, 2010) or simultaneous bi-hemispheric combined stimulation (Lindenberg et al., 2010) have provided promising results supporting the notion of pathological interhemispheric competition and its role in rehabilitation (Schulz et al., 2013). Yet future studies will need to resolve controversies related to the individual tailoring of NBS, i.e., patient stratification according to type, extent of clinical deficit, recovery stage, lesion location and size, patient age, and gender (Hummel et al., 2008). Similarly, it has also been suggested that adjunctive NBS should be combined with simultaneous physiological input (sensory or physiotherapy). Alternatively, one can address the experimental basis of NBS to resolve these controversies. This would imply understanding the underlying mechanism to define the optimal stimulus parameters (Hummel et al., 2008). Here we argue, that the optimal stimulus parameters can be estimated relative to a state-variable of interest (e.g., pinch-strength or some brain-state of motor function) and that the underlying mechanism can be adequately described by noisy and oscillatory neural processes. The hypothesis is that optimal patterning of NBS to drive noisy and oscillatory brain rhythms, i.e., stimulation tailored on-the-fly, are essential for recovery maximization.

It is presently unclear if this might involve potentiating the μ rhythm (Jackson and Zimmermann, 2012), γ rhythm (Schoffelen et al., 2005), or multiplex brain-states reflecting function, dysfunction, or recovery in the central motor system (Brown and Marsden, 2001; Thut and Pascual-Leone, 2010). Despite positive results in a large body of pilot studies, clinical class I evidence has recently been provided in two independent studies that generic rTMS stimulation did not suffice to maximize recovery in stroke patients (Kakuda et al., 2012; Talelli et al., 2012). Conversely, patterned stimulation is providing information about finger-prints in cognitive processes (Thut et al., 2011), brain-computer interface decoding is advancing neurorehabilitation (Jackson and Zimmermann, 2012), deep-brain stimulation must control for frequency dependent side effects (Fogelson et al., 2005), and closed-loop stimulation successfully controls epilepsy in the rat (Berenyi et al., 2012). Finally the proposed framework is in line with a general notion that NBS (homeostatic priming) in combination with peripheral input (inducing a recovery related brain-state) is likely best suited to successfully maximize the speed and duration of spontaneous restitution (Chen et al., 2002; Nowak et al., 2010; Kakuda et al., 2012; Schulz et al., 2013). Thus, in review of a wide variety of data we find that patterned NBS is a promising procedure that could account for many brain-state dependent factors responsible for variability in previous recovery maximization studies. The challenge is to get the right patterning.

### Perspective

**Figure 2:**
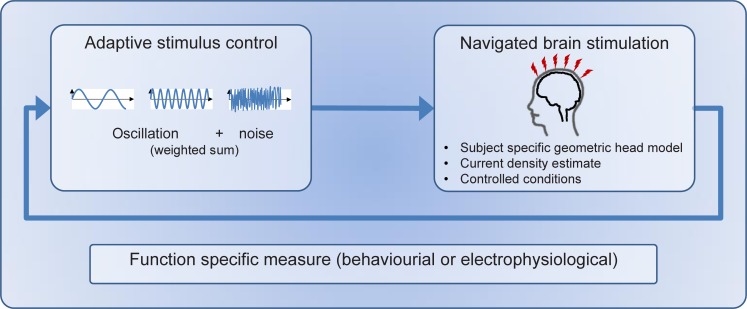
**In order to formulate a closed-loop approach for different stimulation paradigms, the following components are needed: (i) a set of stimulation parameters, common to all methods (e.g., stimulus strength per frequency), (ii) a measure of success (e.g., cortico-muscular coherence), and (iii) a means of adapting the stimulation parameters utilizing this measure**.

Since it is unclear how exactly NBS affects cortical structures and how to correctly pattern NBS to modify a given subject-function specific “finger-print,” we propose a promising, yet simple solution to this seemingly complex problem: i.e., a stimulation algorithm that quickly adapts stimulation patterning online in a closed-loop procedure utilizing a predefined state-variable (behavioral or electrophysiological) associated with motor recovery – similar to a simple brain-computer interface (Jackson and Zimmermann, 2012) (see Figure 2). This allows not only for the elimination of ill-defined prerequisites, but – by careful analysis of stimulus characteristics and their effects on the motor performance – it also provides the enticing perspective to obtain information about the temporo-spatial dynamics of brain-states and their role in motor control, plasticity, and restitution in stroke patients.

## Conflict of Interest Statement

The authors declare that the research was conducted in the absence of any commercial or financial relationships that could be construed as a potential conflict of interest.

## Footnotes

^1^Brain states are understood to reflect instantaneous and transient couplings of neural networks due to phase-locked coupling of bandwidth-confined coherent oscillations.

^2^The sensorimotor sampling loop is understood to collect sensory data necessary for guiding movements (MacKay, 1997).

^3^Frequency-division multiplexing in neural networks describes the communication of several signals over a single medium by sending in several distinct frequency ranges, see, e.g., Thut et al. (2011).

^4^Brain-states are reproducible function specific brain patterns. But, no two subject’s brain patterns are identical. Finger-prints, highly reproducible subject specific entities, are understood to circumscribe the subject specificity of brain-states.
